# Comparison of Polyethylene Glycol versus Lactulose Oral Solution for Bowel Preparation prior to Colonoscopy

**DOI:** 10.1155/2019/2651450

**Published:** 2019-04-11

**Authors:** Chun-Xia Li, Yan Guo, Yang-Jie Zhu, Jian-Ru Zhu, Qian-Song Xiao, Dong-Feng Chen, Chun-Hui Lan

**Affiliations:** Department of Gastroenterology, Daping Hospital, Army Medical University, 10 Changjiang Branch Rd., Chongqing 400042, China

## Abstract

**Objective:**

This study was conducted to compare a lactulose oral solution with a polyethylene glycol (PEG) formulation for colonoscopy preparation using the following metrics: quality of cleansing, colonoscopy outcomes, patient/physician satisfaction, and patient tolerability.

**Methods:**

The enrolled patients were randomly divided into two groups and received a single 2 L dose of either PEG (PEG group) or lactulose (Lac group). The Boston Bowel Preparation Scale (BBPS) was used for assessing the cleansing quality of the bowel preparations. Patient tolerability and adverse events were obtained through the completion of questionnaires.

**Results:**

The lactulose oral solution showed superior bowel cleansing compared to PEG, as evidenced by higher BBPS scores in the Lac group for all segments of the colon (*P* < 0.05). The detection rates of polyps and intestinal lesions in the Lac group (30.68% and 36.36%, respectively) were significantly higher than those in the PEG group (12.50% vs. 13.63%, respectively). For the degree of satisfaction, the Lac group had significantly higher scores compared to the PEG group, as evaluated by both the patients and endoscopist. PEG was associated with an increased incidence of nausea. There were no statistical differences between the groups in terms of vomiting, abdominal pain or fullness, dizziness, unfavorable palatability, dry mouth, palpitation, tinnitus, and tongue numbness.

**Conclusion:**

A single 2 L dose of a lactulose oral solution had higher efficacy, improved tolerability, and acceptable safety for bowel preparation when compared to the same volume of PEG. Thus, a lactulose oral solution may be a potential bowel-cleansing option for colonoscopy preparation.

## 1. Introduction

A colonoscopy is a minimally invasive procedure that is widely used for the diagnosis and treatment of colonic disorders. During a colonoscopic examination, visualization of the mucosa of the entire large intestine and distal terminal ileum is usually possible. Bowel preparation is an essential part of a successful colonoscopy. Inadequate bowel preparation is negatively associated with screening or surveillance outcomes, resulting in misdiagnosis or delayed diagnosis. Inadequate bowel preparation may also lead to suboptimal colonoscopy efficiency, prolonged cecal intubation time, decreased cecal intubation success rates, increased withdrawal time, and additional costs [1, 2]. Ideally, the bowel-cleansing agent should be highly effective and safe and should not cause damage to the intestinal mucosa. Polyethylene glycol (PEG) solution has become the preferred bowel-cleansing agent due to its proven safety and efficacy [3]. Evidence has shown that a split-dose preparation or a single low-volume PEG dose (2 L), compared to receiving a single high-volume PEG dose (4 L), is preferred by patients due to fewer side effects and improved tolerability [4–6]. However, PEG is poorly tolerated by some patients due to its unfavorable palatability.

Lactulose oral solution is used to treat symptoms of constipation. It tastes sweet and has no obvious gastrointestinal side effects. The combined application of lactulose oral solution and PEG has been proven effective for colonoscopy bowel preparation in patients with constipation [7]. However, there is a lack of research describing bowel cleansing and colonoscopy outcomes using lactulose oral solution alone. In this study, we compared the use of a lactulose oral solution (2 L) with a PEG formulation (2 L) for colonoscopy preparation using the following metrics: quality of cleansing, colonoscopy outcomes, patient/physician satisfaction, and patient tolerability.

## 2. Materials and Methods

### 2.1. Study Design

This study was a prospective, randomized, single-blind clinical trial (clinical trial registration number: ChiCTR1800015940). The sample size calculation performed prior to the study revealed that 73 subjects would be required in each group to detect a two-sided difference in treatment success between the two groups, with *α* = 0.01, 1–*β* = 0.95, and an equal size (1 : 1 ratio) for each group. The final sample size comprised at least 81 subjects in each group, assuming 10% missing data. The study was approved by the Institutional Review Board and Human Ethics Committee of the Third Affiliated Hospital, Army Medical University, and was conducted in compliance with the principles in the Declaration of Helsinki. All subjects enrolled provided written informed consent.

### 2.2. Study Subjects

**Figure 1:**
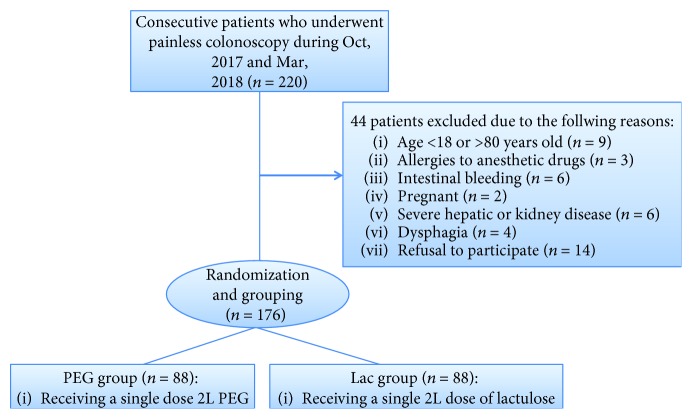
The flow chart of the study.

A total of 220 patients who underwent a colonoscopy during the period from Oct 2017 to Mar 2018 at the Gastroenterology Endoscopy Center in the Third Affiliated Hospital, Army Medical University, were reviewed. Patients were eligible if they (1) were outpatients requiring bowel preparation for colonoscopy examination; (2) were aged 18-80 years old, male or female; (3) had a physical status class I-III according to the American Society of Anesthesiologists (ASA); and (4) were willing to sign a consent form and able to complete the questionnaires. Exclusion criteria included the presence of (1) allergy to anesthetic drugs; (2) severe heart, lung, liver, or kidney diseases, metabolic disorders (including diabetes), or electrolyte disturbance; (3) intestinal perforation, obstruction, or bleeding; (4) pregnant or lactating women; (5) mental disorders; and (6) dysphagia. The flow chart of the study is presented in Figure 1.

### 2.3. Randomization and Grouping

The enrolled patients were randomly divided into two groups at a ratio of 1 : 1 by a random number table in an envelope. In a single dose, patients were given 2 L of PEG (PEG group) or 2 L of lactulose (Lac group) in the morning for colonoscopy preparation. The researchers who generated the random number table did not participate in the subsequent experiments.

### 2.4. Bowel Preparation

Polyethylene glycol electrolyte powder (Wanhe Pharmaceutical Co. Ltd., China) contained PEG-4000 (118 g), sodium sulfate (11.37 g), sodium bicarbonate (3.37 g), sodium chloride (2.93 g), and potassium chloride (1.48 g), which were reconstituted in water (2 L). The lactulose solution was purchased from Abbott Healthcare Products B.V. (Duphalac, Olst, the Netherlands; 200 mL/bottle).

Patients were instructed to eat low-fiber, low-residue, and easily digestible foods the day before the procedure [8]. Foods such as fruits, vegetables, cereals, fried or spicy items, chocolate, coffee, and tea were not allowed [9]. Patients were allowed an early light dinner.

Patients received 2 L of the PEG solution (PEG group) or 200 mL of lactulose followed by an additional 2 L of water (Lac group), to consume orally, at 5:00 AM on the day of the colonoscopy, at a rate of 250 mL every 10-15 minutes within a period of 2 hours. All enrolled patients were given instruction verbally or with an education pamphlet regarding the administration protocol of the bowel-cleansing agents and dietary restrictions. The nurses involved did not participate in subsequent colonoscopy procedures.

### 2.5. Endoscopic Procedure

All colonoscopies were scheduled between 8:00 AM and 11:00 AM. Patients received midazolam (0.01-0.02 mg/kg), remifentanil (0.4 *μ*g/kg), and propofol (1-2 mg/kg) intravenously to induce anesthesia. The patients were given nasal oxygen supplementation with a flow rate of 3 L/min. Patients were lying in a left lateral decubitus position during colonoscopy and maintained in deep sedation with a Ramsay sedation score of 4 or greater. The blood pressure, heart rate, and oxygen saturation of patients were monitored using a multifunctional monitoring system. All colonoscopies were performed using CLV-260SL (Olympus Medical Systems, Tokyo, Japan) and the Fujinon 4400 electronic video endoscope system (Fujinon Corporation, Tokyo, Japan). Upon completion of the colonoscopy, the patients were taken to a resuscitation room and were not allowed to leave until they had fully recovered from sedation. All patients were advised to consume liquid food at a low temperature, two hours after the procedure.

### 2.6. Assessment of Bowel Cleansing

The colonoscopy was performed by an experienced endoscopist who had previously completed more than 1000 colonoscopies. Bowel cleansing was scored by the endoscopist performing the colonoscopy. The Boston Bowel Preparation Scale (BBPS) was used for assessing the cleansing quality of the bowel preparations [10] (Table 1). For all three sections of the large intestine, cleansing was assessed and scored from 0 to 3 and these segment scores were summed for a total BBPS score ranging from 0 to 9. If colon segments were not seen due to inadequate cleansing or bowel distortion, these segments were assigned a score of 0. Adequate bowel preparation was defined as total BBPS ≥ 6 with each individual segment score ≥ 2.

### 2.7. Data Collection

Demographic and baseline patient characteristics, including age, gender, body mass index (BMI), constipation, smoking, drinking, and surgical history, were collected.

Cecal intubation time, colonoscopy withdrawal time, and polyp detection rate were recorded by the endoscopist performing the colonoscopy. Cecal intubation time was defined as the time required from the introduction of the colonoscope to the point where the base of the cecum was reached. Colonoscopy withdrawal time was defined as the length of time taken to remove the colonoscope once the cecum or terminal ileum was reached, including the duration of biopsy and endoscopic treatment. Polyp detection rate was defined as the percentage of procedures where at least one polyp was detected.

### 2.8. Assessment of Patient Tolerability and Adverse Events

Patient tolerability and adverse events of the bowel preparation were obtained with questionnaires completed by the patients before the colonoscopy procedure, which recorded the presence or absence of nausea, vomiting, abdominal pain or fullness, dizziness, palpitation, or other adverse events. The palatability of the bowel-cleansing agents was also recorded.

### 2.9. Satisfaction Assessment

The degrees of satisfaction were evaluated by patients for the entire bowel preparation process as well as by the endoscopist for the endoscopic procedure, using a 10-point scale with 0 being the least satisfied and 10 being the most satisfied.

### 2.10. Statistical Analyses

SPSS for Windows (version 19.0, SPSS Inc., USA) was used for statistical data analysis. Quantitative data were expressed as mean ± standard deviation (SD) and compared using the independent-samples *t*-test. Categorical data are presented as absolute values and percentages and compared using the chi-squared test or Fisher's exact test where appropriate. All tests were two sided, and a *P* value of <0.05 was considered statistically significant.

## 3. Results

### 3.1. Patient Characteristics

The final study population included 176 patients (84 males and 92 females) with a mean age of 49.6 ± 12.0 years old. There were 88 patients in each group, and no significant differences were found in age, gender, BMI, education, smoking or drinking habits, constipation, and history of abdominal disease or surgery (all *P* > 0.05, Table 2).

There were no significant differences in the proportion of patients who strictly adhered to the administration time requirement (at a rate of 250 mL every 10-15 minutes within a period of 2 hours) and drinking-water requirement (≥2L; all *P* > 0.0.5). Also, no statistically significant differences were found between the two groups with respect to the proportion of patients needing flushing during the colonoscopy procedure, mean cecal intubation time, rate of cecal intubation success, or mean anesthetic dosage (propofol; all *P* > 0.05) as shown in Table 3.

### 3.2. Efficacy of Bowel Cleansing Assessed with BBPS Score

The comparative efficacy of the bowel preparation was evaluated using the BBPS scoring system. The Lac group had superior bowel cleansing compared to the PEG group, as evidenced by the higher BBPS scores for all segments of the colon (all *P* < 0.05, Table 4).

### 3.3. Detection of Polyps and Intestinal Diseases

The detection rates of polyps and intestinal diseases in the Lac group (30.68% and 36.36%, respectively) were significantly higher than those in the PEG group (12.50% and 13.63%, respectively; both *P* < 0.05) as shown in Table 5.

### 3.4. Satisfaction of Patients and Endoscopist

Patients in the Lac group had significantly higher satisfaction scores than those of the PEG group, as evaluated by both patients and the endoscopist (both *P* < 0.05; Table 6).

### 3.5. Adverse Events and Palatability

The administration of the lactulose oral solution, as compared with the PEG solution, was associated with a lower incidence of nausea (23.81% in the Lac group vs. 58.54% in the PEG group, *P* = 0.008; Table 7). Two patients (4.88%) in the PEG group complained of the unfavorable palatability, but there were no complaints from patients in the Lac group. However, the difference did not achieve statistical significance (*P* = 0.497). There were no statistical differences between the groups in terms of vomiting, abdominal pain or fullness, dizziness, dry mouth, palpitation, tinnitus, and tongue numbness (all *P* > 0.05). However, there was one case of tinnitus and one case of tongue numbness in the Lac group, but none were observed in the PEG group.

## 4. Discussion

Adequate bowel preparation is required for diagnostic accuracy and therapeutic safety for colonoscopy procedures [11]. In approximately 10-20% of colonoscopies, intubation of the cecum may be difficult [12], which can result in a failure to detect polyps or lesions and increase the risk of procedure-related complications. Although there are various reasons for colonoscopy failure, inadequate bowel preparation is considered a substantial cause for decreased colonoscopy effectiveness [13]. It has been reported that bowel preparation is inadequate in almost one-quarter of patients undergoing colonoscopy [14]. The qualities of an ideal bowel-cleansing agent include confirmed safety with minimal discomfort, high efficacy, high rate of patient compliance, and inexpensive price.

PEG is a nonabsorbable isoosmotic solution, which passes through the bowel without any net absorption or secretion. Currently, PEG-based regimens remain the first recommendation for bowel preparation. PEG causes no significant change in weight, vital signs, or serum electrolytes, and it is also relatively safe for patients with an electrolyte imbalance or those with advanced liver, heart, or renal disease [9]. Unfortunately, it is poorly tolerated in 5-15% of patients due to the large volume (4 L) required and poor palatability of the solution [15]. Lactulose is a synthetic disaccharide that is widely used for the treatment of constipation and hepatic encephalopathy because of its efficacy and good safety profile. It cannot be broken down nor be absorbed by the small intestine due to a lack of lactulose-specific enzymes. After entering the colon, lactulose is broken down by *β*-galactosidase produced by bifidobacteria to provide a carbon source for the growth of bifidobacteria. It acidifies the intestinal environment and inhibits the growth of harmful bacteria [16]. The mechanism of action of lactulose may be due to its ability to remain unchanged in the lower gastrointestinal tract, where it increases the retention of water and electrolytes by its osmotic effect; it also enhances bowel motility stimulated by organic acid to which lactulose is broken down by enterobacteria [17]. In this study, we evaluated the efficacy of a lactulose solution for bowel cleansing. The lactulose solution showed a superior bowel cleansing capacity compared to PEG, since the Lac group had higher BBPS scores in all colon segments compared to the scores from the PEG group. Consistently, lactulose facilitated the detection of polyps and intestinal lesions, which validated the use of the lactulose solution for bowel preparations.

In this study, no significant difference was observed in the proportion of patients needing flushing during the colonoscopy procedure between the PEG and Lac groups. Neither PEG nor lactulose itself is capable of removing bubbles. Thus, there might be a large amount of foam residue in the gut after bowel preparation, and as a result additional flushing is required during the procedure. These data indicated a similar effect of lactulose and PEG on the removal of foam as a bowel-cleansing agent.

Relative to PEG, lactulose tastes sweeter and is well tolerated by patients. In this study, there were no patients in the Lac group who did not ingest the preparation due to poor taste. Also, patients were more satisfied with the lactulose solution for the entire bowel preparation process, likely due to its better taste. In contrast, two patients in the PEG group complained of unfavorable palatability, and one of them vomited as a result of intolerability. PEG was associated with a higher incidence of nausea (58.54%) after administration, which might be related to its unfavorable palatability. Adverse reactions in the Lac group were mainly gastrointestinal symptoms, such as nausea, vomiting, abdominal pain, and abdominal fullness. Importantly, patients who were given the lactulose solution were more likely to develop abdominal fullness, as compared to PEG (16.67% vs. 4.88%). Since the body is able to absorb only a small amount of lactulose, a patient would feel a sense of abdominal fullness and maybe even experience vomiting if substantial amounts of the lactulose solution were ingested within a short period of time. There were similar overall incidences of adverse reactions in both groups, suggesting that the lactulose solution is safe to use for bowel preparations. However, the long-term safety profile of lactulose needs further investigation.

In conclusion, this study demonstrated the higher efficacy, improved tolerability, and acceptable safety of a 2 L lactulose oral solution for bowel preparation as compared with the same volume of a PEG solution. Thus, the lactulose oral solution may be a potential bowel-cleansing agent for use in colonoscopy preparation.

## Figures and Tables

**Table 1:** The Boston Bowel Preparation Scale.

Points	Description
0	Unprepared colon segment with mucosa not seen due to solid stool that cannot be cleared
1	Portion of mucosa of the colon segment seen, but other areas of the colon segment not well seen due to staining, residual stool, and/or opaque liquid
2	Minor amount of residual staining, small fragments of stool, and/or opaque liquid, but mucosa of colon segment seen well
3	Entire mucosa of colon segment seen well with no residual staining, small fragments of stool, or opaque liquid

**Table 2:** Patient characteristics.

	PEG (*n* = 88)	Lac (*n* = 88)	*P* value
Age (years)	49.63 ± 11.98	50.15 ± 10.66	0.754
Male, *n* (%)	48 (54.6)	36 (40.9)	0.07
Bodyweight (kg)	62.52 ± 11.48	59.44 ± 9.76	0.057
Height (cm)	162.84 ± 8.01	160.65 ± 6.91	0.053
Education (high school and above), *n* (%)	40 (45.5)	31 (35.2)	0.167
BMI (kg/m^2^)	23.46 ± 3.27	22.97 ± 3.11	0.312
Smoking, *n* (%)	20 (22.7)	20 (22.7)	1.000
Drinking, *n* (%)	22 (25.0)	20 (22.7)	0.724
History of abdominal surgery, *n* (%)	21 (23.9)	18 (20.5)	0.586
Constipation, *n* (%)	23 (26.1)	24 (27.3)	0.865
Abdominal disease^∗^, *n* (%)	27 (30.7)	16 (18.2)	0.054

^∗^ included inflammatory bowel disease, abdominal tumor, gynecological tumor and inflammation, mesenteric tuberculosis, and intestinal tuberculosis.

**Table 3:** Bowel preparation characteristics and colonoscopy results.

	PEG (*n* = 88)	Lac (*n* = 88)	*P* value
Meeting administration time requirement, *n* (%)	83 (94.3)	77 (87.5)	0.190
Meeting drinking-water requirement (≥2 L), *n* (%)	76 (86.4)	77 (87.5)	0.823
Cecal intubation time (s)	303.85 ± 196.19	316.92 ± 238.56	0.692
Cecal intubation success, *n* (%)	85 (96.6)	86 (97.7)	1.000
Patients needing flushing, *n* (%)	23 (26.1)	27 (30.7)	0.504
Propofol dosage (ml)	15.58 ± 5.83	14.51 ± 3.02	0.129

**Table 4:** Efficacy of bowel cleansing assessed with BBPS score.

	PEG (*n* = 88)	Lac (*n* = 88)	*P* value
Right colon	2.14 ± 0.66	2.51 ± 0.70	0.001
Transverse colon	2.52 ± 0.68	2.75 ± 0.57	0.017
Left colon	2.23 ± 0.69	2.69 ± 0.49	0.001
Entire colon	6.88 ± 1.78	7.95 ± 1.40	0.001

**Table 5:** Detection of polyp and intestinal lesions.

	PEG (*n* = 88)	Lac (*n* = 88)	*P* value
Polyp, *n* (%)	11 (12.5)	27 (30.7)	0.003
Intestinal lesions^∗^, *n* (%)	12 (13.6)	32 (36.4)	0.0013

∗ includes intestinal polyps, intestinal adenomas, colon cancer, inflammatory bowel disease, colon melanosis, radiation enteritis, and colonic submucosal lesions.

**Table 6:** The degrees of satisfaction evaluated by patients and endoscopist.

	PEG (*n* = 88)	Lac (*n* = 88)	*P* value
Patient satisfaction	8.36 ± 1.67	8.91 ± 1.34	0.018
Endoscopist satisfaction	7.74 ± 1.43	8.57 ± 1.01	0.001

**Table 7:** Comparison of adverse events and tolerability, *n* (%).

	PEG (*n* = 88)	Lac (*n* = 88)	*P* value
Nausea	24 (58.5)	10 (23.8)	0.008
Vomiting	8 (19.5)	16 (38.1)	0.079
Abdominal pain	1 (2.4)	2 (4.8)	0.623
Abdominal fullness	2 (4.9)	7 (16.7)	0.168
Dizziness	2 (4.9)	4 (9.5)	0.682
Unfavorable palatability	2 (4.9)	0	0.497
Dry mouth	2 (4.9)	1 (2.4)	0.623
Palpitation	2 (4.9)	1 (2.4)	0.623
Tinnitus	0	1 (2.4)	1.000
Tongue numbness	0	1 (2.4)	1.000
Total	36 (40.9)	42 (47.7)	0.363

## Data Availability

The data used to support the findings of this study are available from the corresponding author upon request.
